# Minimally invasive internal fixation for unstable pelvic ring fractures: a retrospective study of 27 cases

**DOI:** 10.1186/s13018-021-02387-5

**Published:** 2021-05-31

**Authors:** Shuang Wu, Jialei Chen, Yun Yang, Wei Chen, Rong Luo, Yue Fang

**Affiliations:** Department of Orthopaedics, West China Hospital, Sichuan University, No.37 Guoxue Lane, Chengdu, 610041 Sichuan People’s Republic of China

**Keywords:** Pelvic fracture, Pelvic ring injury, INFIX, Minimally invasive, Internal fixation

## Abstract

**Background:**

This study aimed to evaluate the radiographic and clinical outcomes of anterior subcutaneous internal fixation (INFIX) with or without posterior fixation for the treatment of unstable pelvic fractures.

**Methods:**

Intraoperative blood loss, operation time, and duration of hospital stay were recorded, and fracture union and postoperative complications were evaluated. The fracture reduction quality was evaluated using the Matta score, pelvic deformity index (PDI), and pubic symphyseal width (PSW). In addition, the functional recovery and general quality of life were evaluated using the Majeed score and the 12-Item Short-Form Survey (SF-12), respectively. Furthermore, sacral nerve injury was evaluated using the Gibbons classification.

**Results:**

Twenty-seven patients (14 males and 13 females) with an average age of 37.4 years were followed up for a mean of 22 months. The average operation time, median intraoperative blood loss, and average duration of hospital stay were 129 ± 47 min, 100 mL, and 22 ± 13 days, respectively. All patients achieved bony union with an average union time of 13.3 weeks. Furthermore, the average PDI and PSW were 0.07 ± 0.04 vs. 0.04 ± 0.03 (*P* = 0.009) and 1.15 ± 1.36 vs. 0.54 ± 0.17 (*P* = 0.048) before and after the operation, respectively. In 78% of the patients, the Matta or Majeed scores were excellent or good. The SF-12 physical and mental health scores were 45.1 ± 10.2 and 53.2 ± 6.3, respectively. Furthermore, one superficial surgical site infection, one loosening of INFIX, one lateral femoral cutaneous nerve irritation, one femoral nerve injury, and two implant discomforts due to the bar were noted. Among five patients with sacral nerve injuries, four were asymptomatic, and one just had paresthesia at the last follow-up.

**Conclusion:**

INFIX with or without sacroiliac screws can achieve satisfactory radiographic and functional outcomes in the treatment of unstable pelvic ring fractures.

**Trial registration:**

ChiCTR2000038812. Registered 04 October 2020. Retrospectively registered.

## Background

Pelvic ring fractures are uncommon, accounting for only 3–8% of adult fractures [1]. However, they often cause considerable mortality at 6–31% [2]. Approximately 50% of the patients have unstable pelvic ring fractures that are often accompanied by hemodynamic instability usually caused by high-energy injuries, which often damage the bone–ligament structures and then cause vertical or rotational instability of the pelvis [3]. Anterior ring provides 30–40% pelvic stability and posterior ring provides 60–70% pelvic stability [4, 5]. Posterior fractures of unstable pelvic ring injuries often require operative fixation because conservative treatment often fails to achieve good outcomes [6]. Moreover, anterior fixation can further improve the biomechanical stability of the pelvic ring.

Because pelvic ring fractures are often complicated with severe multiple traumas, no gold standard exists, although there are several available therapeutic methods. Posterior ring fractures can be treated with open reduction and internal fixation (ORIF) or minimally invasive surgery including locking compression plates, reconstruction plates, spinopelvic fixation, and percutaneous sacroiliac (SI) screws. Furthermore, anterior pelvic ring injuries are mainly fixed by external fixation (EXFIX), ORIF, and minimally invasive surgery such as subcutaneous pedicle screw–rod system (INFIX), pubic ramus screw, and pelvic bridge.

Pelvic EXFIX is usually employed for the fixation of anterior pelvic ring fractures and emergency treatment of pelvic fractures. However, several complications exist (e.g., pin tract infection, osteomyelitis, screw loosening, reoperation, limited patient activity, and difficulty in nursing care). Moreover, patients with ORIF often experience more operative trauma, postoperative complications, and long rehabilitation time [7, 8]. In recent years, INFIX has been one of the novel minimally invasive internal fixations because it can help achieve good clinical and radiographic results and reduce the incidence of the abovementioned complications [9]. INFIX is still not accepted worldwide and there is a need to conduct more research to evaluate the therapeutic effect of INFIX. Thus, this study reviewed the radiographic and clinical outcomes and complications of 27 patients with unstable pelvic ring fractures managed with anterior INFIX with or without posterior SI screws.

## Methods

This study was approved by the Biomedical Research Ethical Committee of West China Hospital of Sichuan University and registered in Chinese Clinical Trial Registry (registration number: ChiCTR2000038812). We reviewed 27 patients who underwent INFIX fixation with or without SI screws in our hospital from June 2016 to October 2019 for the treatment of unstable pelvic fractures. The inclusion criteria were age (≥ 16 years), unstable pelvic ring injury (Tile classification types B1–B3 or type C), and INFIX management with or without SI screws. The exclusion criteria were open pelvic fracture, patients with severe osteoporosis, and soft tissue infection or fracture at the area of screw placement. Moreover, patients who underwent pelvic EXFIX for emergency treatment owing to hemodynamic instability were not excluded. Finally, 27 patients (14 males and 13 females) with an average age of 37.4 years were enrolled and followed up for 22 months (range, 12–34) based on the above criteria.

The baseline data of patients were extracted from the electronic medical record system. The demographic data included age, gender, and body mass index (BMI). The basic clinical data included injury severity score (ISS), fracture classification (Tile and Young–Burgess classification), injury mechanism, fracture side of the anterior ring, time to operation, need for intensive care unit (ICU), length of ICU stay, and follow-up time. According to ISS, all patients were divided into ISS < 25 and ISS ≥ 25 subgroups [10]. The types of pelvic fractures were classified by two orthopedic surgeons (SW and JLC) based on the radiological data. The third senior orthopedic surgeon (YF) was invited to discuss if disagreements arise.

**Fig. 1:**
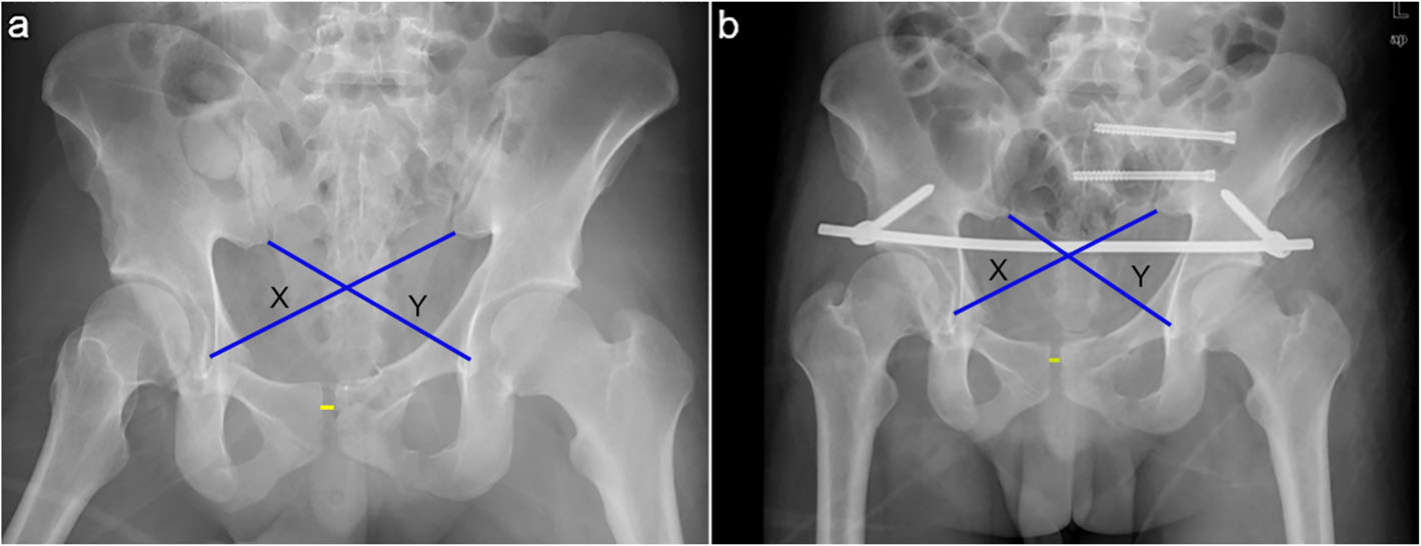
Measurement of pelvic deformity index (PDI) and pubic symphyseal width (PSW). **a** Preoperative AP film. **b** Postoperative AP film. X or Y is the diagonal length from the inferior SI joint (iliac side) to the inferior aspect of the teardrop on an AP film. PDI = absolute (X − Y)/(X + Y)

Operation-related indices included the operation time, blood loss, duration of hospital stay, need for posterior fixation, need for additional anterior fixation, postoperative complications, and INFIX removal. Operation time was defined as the total time between sterilization and the end of surgical suture. The clinical outcomes included fracture reduction, radiographic union, and functional evaluation. The pelvic fracture reduction quality was evaluated using the Matta score, pelvic deformity index (PDI), and pubic symphyseal width (PSW). The Matta score is graded based on the maximum displacement of the fracture on the anteroposterior, inlet, and outlet views of the pelvic X-ray as excellent (≤ 4 mm), good (4–10 mm), fair (10–20 mm), and poor (> 20 mm) [11]. The PDI cross-measurement method was first described by Keshishyan et al. [12] and then employed to evaluate the pelvic asymmetry of unstable pelvic injuries in children by Smith et al. [13]. Recently, Vaidya et al. used PDI and PSW to evaluate the reduction of unstable pelvic ring fractures treated with INFIX [14]. Figure 1 presents the PDI calculation. Consequently, PSW was the largest width of the symphysis pubis, as described by Vaidya et al. [14]. Fracture union was mainly evaluated based on callus growth on three views of pelvic X-ray. The Majeed score was utilized to evaluate pelvic functional recovery as excellent (85–100), good (70–84), fair (55–69), and poor (< 55) [15]. The 12-Item Short-Form Survey (SF-12) score was used to evaluate the general health-related quality of life [16]. Moreover, the mental component score (MCS) and physical component score (PCS) were calculated from SF-12 based on Ware et al.’s manual [17]. Sacral nerve injury was also evaluated using the method described by Gibbons et al. [18].

**Fig. 2:**
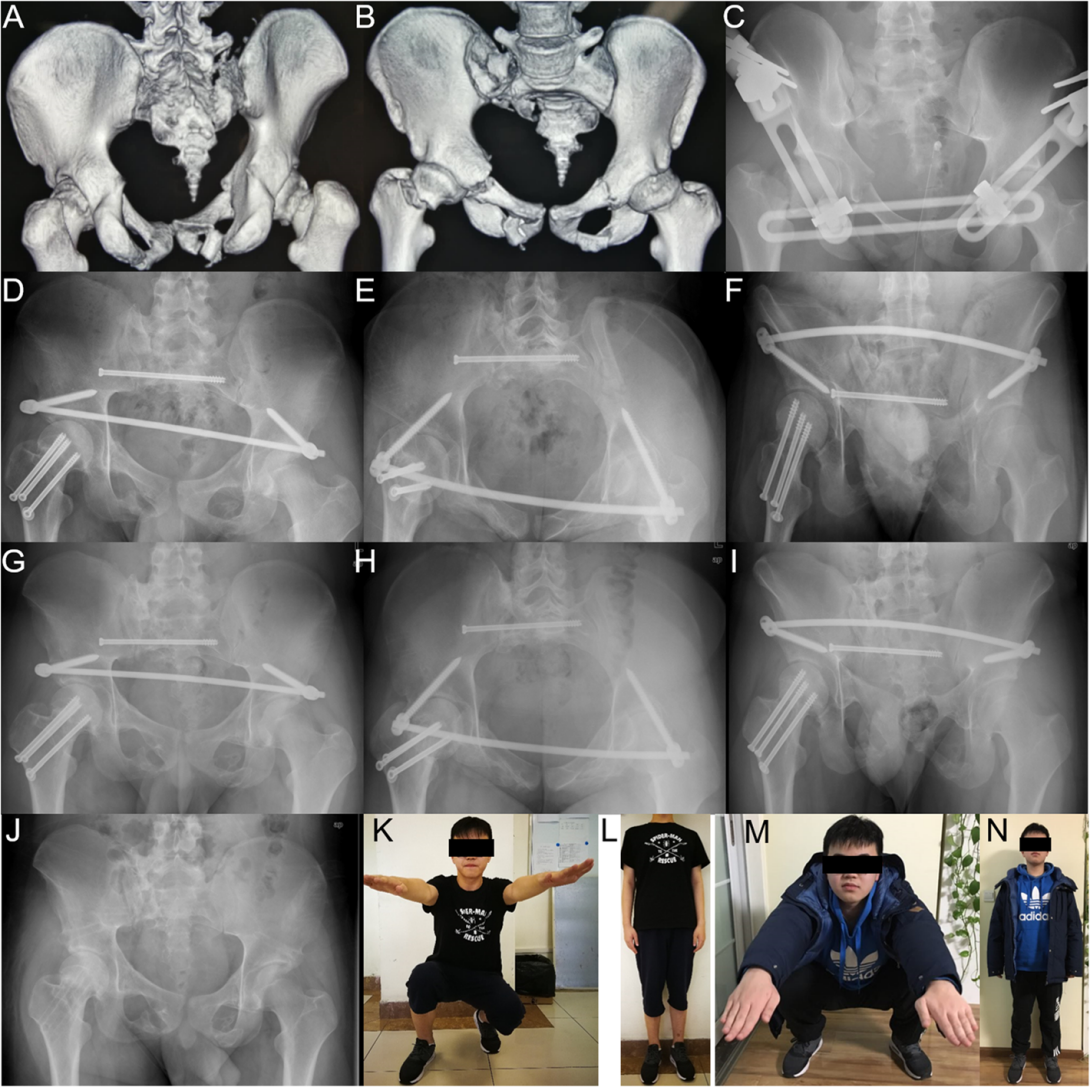
A 17-year-old man with anterior and posterior pelvic ring fractures and right femoral neck fracture. **a, b** Preoperative 3D CT reconstruction of pelvis. **c** AP pelvic film after external fixation. **d**–**f** Postoperative pelvic films (AP, inlet and outlet views) showing fracture union at 6 months. **g**–**i** Postoperative pelvic films (AP, inlet and outlet views) at 12 months. **j** AP pelvic film after implant removal at 12 months. **k**, **l** Walking and standing were not limited while right hip flexion was limited at 6 months follow-up. **m**, **n** Hip Flexion was more than 90°and Majeed score was excellent at 12 months follow-up

All operations were performed by an experienced orthopedic surgeon (YF), who has been practicing for > 20 years. Before the operation, imaging examinations, including three X-ray views, computed tomography (CT), and three dimensional (3D) reconstruction of the pelvis, were conducted to evaluate the fracture and estimate the length of the connecting rod of INFIX. Posterior pelvic ring fractures were reduced using standard sacroiliac reduction techniques and percutaneously fixed with one to two sacroiliac screws (diameter, 6.5 mm) [19, 20]. Anterior ring fractures were reduced and fixed with INFIX (Medtronic, Memphis, TN, USA) based on the methods described by Müller et al. and Vaidya et al. [21, 22]. Furthermore, INFIX included bilateral polyaxial pedicle screws (diameter, 7.5 mm; length, 60–80 mm) and a titanium rod (5.7 mm). Additional plates, Kirschner wires, or screws were utilized to strengthen anterior fixation for open-book fractures or comminuted fractures of the anterior ring (Fig. 2).

According to patient status, non-weight-bearing exercise on the bed was encouraged for 4 weeks following the operation. Patients with type B and C fractures were allowed to bear partial weight with crutches at 4–10 and 4–12 weeks following surgery, respectively. Then patients were allowed to bear full weight after achieving radiographic union. All patients were recommended to visit doctors for the evaluation of fracture healing and postoperative complications at 1, 2, 3, 6, and 12 months following the operation. At the last outpatient follow-up, all patients were invited to complete the Majeed and SF-12 questionnaires, and a researcher (YY) who was never involved in the diagnosis, treatment, and follow-up of patients evaluated the quality of fracture reduction using the Matta score, PDI, and PSW.

### Statistical analysis

Statistical analysis was conducted using the Statistical Package for the Social Sciences, version 21 for windows (IBM Corp. Armonk, NY, USA). Descriptive data were expressed as mean ± SD, median and interquartile range (IQR), exact values (*n*), percentage (%), and range. Two independent samples *t* test or Mann–Whitney *U* test was employed to compare the difference in the variables between the two groups. *P* < 0.05 was considered statistically significant.

## Results

The baseline data of this study are presented in Table 1. The predominant injury mechanism was traffic accident injury (14 cases), followed by falling from a height (12 cases) and then heavy object injury (1 case). The ISS median was 29 (range, 14–41). Furthermore, 5 patients were emergently treated in the ICU at an average time of 11.4 ± 2.5 days (range, 7–13). The mean time to surgery was 10.6 ± 6.8 days (range, 1–26). According to the Tile classification, 2 type B fractures (B3.1, 1; B3.2, 1) and 25 type C fractures (C1.1, 4; C1.2, 3; C1.3, 15; C2, 2; and C3, 1) existed. According to the Young–Burgess classification, 6 anteroposterior compression injuries, 12 lateral compression injuries, 7 vertical shear injuries, and 2 complex pelvic injuries existed.

**Table 1:** Demographical characteristics and clinical data of 27 patients

Variables	(*n* = 27)	Range/percent
Demographics		
Age (year, mean ± SD)	37.4 ± 14.4	16–63
Male, *n*	14	51.9%
BMI (kg/m^2^, mean ± SD)	23.3 ± 3.0	17.6–30.1
ISS, median (IQR)	29 (17–34)	14–41
ISS < 25	8	29.6%
ISS ≥ 25	19	70.4%
Fracture side, *n*		
Right	5	18.5%
Left	6	22.2%
Both sides	16	59.3%
Injury type, *n*		
Fall from height	12	44.4%
Traffic accident	14	51.9%
Heavy object injury	1	3.7%
Tile classification, *n*		
Type B3.1	1	3.7%
Type B3.2	1	3.7%
Type C1.1	4	14.8%
Type C1.2	3	11.1%
Type C1.3	15	55.6%
Type C2	2	7.4%
Type C3	1	3.7%
Young-Burgess classification, *n*		
APC	6	22.2%
LC	12	44.4%
VS	7	25.9%
CM	2	7.4%
Time to surgery (day, mean ± SD)	10.6 ± 6.8	1–26
Need for ICU on arrival, *n*	5	18.5%
Duration of ICU, day, mean ± SD	11.4 ± 2.5	7–13

*SD* standard deviation, *BMI* body mass index, *ISS* injury severity score, *IQR* interquartile range, *APC* anteroposterior compression, *LC* lateral compression, *VS* vertical shear, *CM* complex mechanism, *ICU* intensive care unit

Operation-related indices are presented in Table 2. The average operation time was 128.8 min (range, 70–265), and the median blood loss was 100 mL (range, 20–400). Moreover, sacroiliac screws were required to fix the posterior ring in 23 patients. The average duration of hospital stay was 22.2 days (range, 6–61).

**Table 2:** Operational records, clinical outcomes, and postoperative complications of 27 patients

Variables	(*n* = 27)	Range/percent
Radiographic union, n	27	100%
Union time, week, mean ± SD	13.3 ± 2.1	9–17
Majeed score, mean ± SD	87.8 ± 13.0	62–100
Excellent, *n*	19	70.4%
Good, *n*	2	7.4%
Fair, *n*	6	22.2%
Poor, *n*	0	0
SF12		
PCS, mean ± SD	45.1 ± 10.2	23.9–56.2
MCS, mean ± SD	53.2 ± 6.3	33.6–62.3
Matta score, *n*		
Excellent	8	29.6%
Good	13	48.2%
Fair	6	22.2%
Poor	0	0
PDI, mean ± SD		
Pre-operation	0.07 ± 0.04	0.01–0.19
Post-operation	0.04 ± 0.03	0–0.10
PSW, cm, mean ± SD		
Pre-operation	1.15 ± 1.36	0.19 6.37
Post-operation	0.54 ± 0.17	0.15 0.86
Operation time, min, mean ± SD	128.8 ± 46.7	70–265
Blood loss, ml, median (IQR)	100 (60–200)	20–400
Hospitalization time, day, mean ± SD	22.2 ± 12.5	6–61
Posterior ring fixation, *n*	23	85.2%
Additional anterior ring fixation, *n*	8	29.6%
Postoperative complications, *n*	9	33.3%
Incision infection	1	3.7%
Implant-related discomfort	2	7.4%
Internal fixation failure	1	3.7%
Femoral nerve palsy	1	3.7%
LFCN irritation	1	3.7%
Pelvic abscess	1	3.7%
Sacrococcygeal pain	2	7.4%
Implant removal, *n*	7	25.9%
Time of implant removal, month	12.9 ± 5.2	3.5–18.5
Follow-up time (month, mean ± SD)	22 ± 8.4	12–34

*SD* standard deviation, *SF12* 12-item short-form health survey, *PCS* physical component score, *MCS* mental component score, *PDI* pelvic deformity index, *PSW* pubic symphyseal width, *IQR* interquartile range, *LFCN* lateral femoral cutaneous nerve

The clinical outcomes are presented in Table 2. Based on Matta’s criteria, an excellent or good rate was 77.8%, and a fair rate was 22.2%. The average PDI and PSW were 0.07 ± 0.04 vs. 0.04 ± 0.03 (*P* = 0.009) and 1.15 ± 1.36 vs. 0.54 ± 0.17 (*P* = 0.048) before and after the operation, respectively. All fractures achieved bony union with the mean union time of 13.3 weeks (range, 9–17). Consequently, INFIX was removed in 7 patients at 12.9 months (range, 3.5–18.5) postoperatively.

The average PCS of SF-12 and MCS were 45.1 ± 10.2 and 53.2 ± 6.3. The average Majeed score was 87.8 ± 13.0 (range, 62–100). Furthermore, the Majeed score was excellent or good in 77.8% of the patients and fair in 22.2% of the patients.

The analysis’ results of the Majeed score- and SF-12-related influencing factors are presented in Table 3. The Majeed score of male patients was significantly higher than that of female patients (*P* = 0.028). Moreover, the Majeed score and PCS of patients with ISS < 25 were higher than those of patients with ISS ≥ 25 (*P* = 0.033 and 0.001, respectively). No other significant influencing factors were observed.

**Table 3:** Analysis of influencing factors of SF-12 and Majeed score

Variables (*n* = 27)	*n*	Majeed score, mean ± SD	*P* value	PCS, mean ± SD	*P* value	MCS, mean ± SD	*P* value
Age							
16 ≤ age < 45	16	91.1 ± 10.8	0.118	48.2 ± 7.9	0.056	54.2 ± 5.0	0.304
45 ≤ age < 65	11	83.1 ± 14.8		40.6 ± 11.9		51.6 ± 7.8	
Gender							
Male	14	93.0 ± 10.5	0.028*	48.3 ± 8.3	0.094	54.6 ± 5.1	0.229
Female	13	82.2 ± 13.4		41.7 ± 11.3		51.6 ± 7.3	
ISS							
ISS < 25	8	94.1 ± 6.4	0.033*	52.4 ± 3.9	0.001*	55.4 ± 3.8	0.237
ISS ≥ 25	19	85.2 ± 14.2		42.0 ± 10.5		52.2 ± 7.0	
Posterior fixator							
Yes	23	87.4 ± 12.9	0.662	45.2 ± 9.9	0.944	53.8 ± 5.3	0.200
No	4	90.5 ± 15.1		44.8 ± 13.6		49.4 ± 10.6	
Anterior fixator							
Yes	8	91.6 ± 11.5	0.331	47.8 ± 9.6	0.391	53.4 ± 8.4	0.901
No	19	86.2 ± 13.5		44.0 ± 10.5		53.1 ± 5.4	
Need for ICU							
Yes	5	89.8 ± 12.9	0.712	45.0 ± 11.1	0.976	50.3 ± 9.9	0.271
No	22	87.4 ± 13.2		45.1 ± 10.3		53.8 ± 5.3	
Fracture side							
Both sides	16	86.8 ± 13.0	0.637	43.6 ± 10.9	0.364	53.5 ± 6.2	0.765
Right/left side	11	89.3 ± 13.3		47.3 ± 9.1		52.7 ± 6.7	

*SD* standard deviation, *PCS* physical component score, *MCS* mental component score, *ISS* injury severity score, *ICU* intensive care unit

*P < 0.05

Postoperative complications are presented in Table 2. Of the patients, one had superficial surgical site infection, one had unilateral lateral femoral cutaneous nerve (LFCN) irritation, one had persistent unilateral femoral nerve (FN) injury, one had implant loosening, and two experienced rod-related discomforts. There were five cases of sacral nerve injury (four patients had paresthesia (grade II) before the operation and gradually fully recovered (grade I) after the operation; one patient had a motor loss (grade III) preoperatively, partially recovered postoperatively, and still had paresthesia (grade II) at the last follow-up).

## Discussion

In the early twenty-first century, INFIX began to be used for anterior pelvic ring fixation in the treatment of unstable pelvic fractures [23]. Vaidya et al. initially believed that patients with obesity having unstable pelvic injuries are the best INFIX indication as EXFIX may compress the abdomen with excessive fat and other operations still have some disadvantages [22]. Moreover, the indications of INFIX have significantly expanded nowadays. INFIX has been employed to treat most unstable pelvic injuries, including type 61-C and 61-B injuries (AO/OTA classification), type B and C fractures (Tile classification), and even some open pelvic fractures [9, 24].

Biomechanical analyses revealed that INFIX could help reduce fracture displacement more than EXFIX and that INFIX was significantly stiffer than EXFIX [25, 26]. Although INFIX was not stiffer than ORIF and could not maintain the reduction of fracture better than ORIF [26], it still had numerous advantages, such as good radiological and functional outcomes, less soft tissue damage, less intraoperative bleeding, short operation time, and low incidence of postoperative complications [27]. Outcomes of INFIX in published studies are shown in Table 4. Furthermore, INFIX was suitable for combined surgery of pelvic and abdominal injuries. In this study, one patient with a type C fracture had a ruptured spleen and underwent emergent exploratory laparotomy and splenectomy. INFIX fixation was performed on day 4 after splenectomy and the wound healed well.

**Table 4:** Characteristics of INFIX and outcomes of published studies using INFIX

Variables	*N*	Country	Screw		Rod d, mm	RBD, mm	Union rate	Majeed score		Matta score Ex/G	Implant removal	
			d, mm	l, mm				Score	Ex/G		Rate	Time, month
Vaidya2012 [28]	91	USA	7.0–8.5	70–110	N/A	N/A	100%	N/A	N/A	N/A	100%	3.0–6.0
Vaidya2012 [22]	22	USA	7.0/8.0	75–110	6.0	15–50	100%	N/A	N/A	N/A	73%	5.4
Müller2013 [21]	31	Germany	6.0/7.0	50–60	6.0	N/A	97%	N/A	65%^a^	N/A	97%	9.4
Hoskins2016 [29]	21	Australia	10.0	100	5.5	N/A	100%	N/A	N/A	N/A	90%	3.6
Shetty2017 [6]	15	India	7.0	70–100	5.7	Deep fascia	100%	93	100%	100%	80%	7.3
Fang2017 [30]	29	China, Germany et al.	5.0–7.0	≥ 55	N/A	15–20	100%	N/A	N/A	N/A	72%	N/A
Dahill2017 [31]	47	UK	5.5–6.5	55–70	N/A	5–10	98%	N/A	N/A	N/A	96%	4.5
Vaidya2017 [14]	83	USA	N/A	N/A	N/A	N/A	100%	79	N/A	N/A	88%	5.3
Wu2018 [32]	23	China	6.5/7.0	60–80	6.0	20	100%	85	91%	87%	N/A	10.0
Li2019 [33]	28	China	6.5/7.0	60–80	6.0	20	100%	81	N/A	86%	79%	5.0
Hua2019 [34]	23	China Germany	7.5	75–110	6.0	Deep fascia	100%	N/A	87%	83%	N/A	N/A
Steer2019 [35]	24	Australia	N/A	N/A	N/A	N/A	100%	80 ^b^	80%^b^	N/A	96%	5.0
Vaidya2019 [36]	39	USA	N/A	N/A	N/A	N/A	100%	N/A	78%	N/A	N/A	3.0–5.0
Ebeed2020 [37]	16	Egypt	6.5/7.3	75–100	6.0	Fascia lata	100%	N/A	100%	94%	N/A	N/A
Du2020 [38]	17	China	6.5	60	N/A	N/A	100%	92	100%	100%	N/A	N/A
Bi2017 [4]	21	China	7.0	80	6.0	N/A	100%	83	90%	N/A	N/A	N/A
Vaidya2017 [27]	24	USA	N/A	N/A	N/A	N/A	100%	84	N/A	N/A	N/A	3.0–5.0
Wang2017 [7]	26	China	N/A	80	N/A	N/A	100%	N/A	81%	N/A	100%	4.3
Ma2019 [8]	62	China	7.0	60–80	N/A	N/A	100%	80	≥ 77%	N/A	N/A	N/A
Yin2019 [39]	35	China	6.5	50	5.5	≥ 20	100%	84	N/A	74%	100%	N/A
Current study	27	China	7.5	60–80	5.7	Deep fascia	100%	88	78%	78%	26%	12.9

*d* diameter, *l* length, *RBD* the distance of rod to bone, *Ex/G* excellent or good

^a^Becken outcome score

^b^Iowa pelvic score

INFIX was a simple and reliable method for treating unstable pelvic ring fractures. In this study, INFIX with or without SI screws could effectively help maintain the reduction based on Matta score, PDI, and PSW, and promote postoperative functional rehabilitation based on Majeed score. According to the standard scoring algorithm (USA) [16], the PCS was lower than that of the normal population (*P* < 0.05), whereas the MCS was higher than that of the normal population (*P* < 0.05), which may be attributed to the fact that most patients had reasonable mental health. ISS and gender may influence Majeed score and SF-12 score (Table 3), whereas Müller et al. found that there was no significant influencing factor [21]. Moreover, the PCS and Majeed score of patients with ISS < 25 were significantly higher than those of patients with ISS ≥ 25, which was consistent with the results of Vaidya et al. [14]. Most of the patients with ISS ≥ 25 were in serious condition and had severe multiple injuries, which often require longer duration of hospital stay for treatment and recovery (25.2 ± 13.5 vs. 14.9 ± 5.0, *P* = 0.047). Contrarily, patients with ISS < 25 could participate in functional rehabilitation earlier owing to requiring shorter duration of hospital stay. Thus, injury severity and time of functional rehabilitation may influence the PCS and Majeed score. Although the Majeed score of male patients was significantly higher than that of female patients at the last follow-up, no statistical difference was observed between the two subgroups in terms of hospitalization time, time to operation, and ISS, which may be owing to the limited sample size.

Postoperative complications of INFIX in published studies are shown in Table 5. There were some common complications related to surgical operation, such as implant loosening, pin tract infection, aseptic loosening, the incidence of which for INFIX was very low compared with that for EXFIX [28]. Failure in INFIX implant only occurred in 0–13% patients [6, 27, 34] and wound problems (e.g., fat liquefaction, incision dehiscence, epidermal infection, and deep infection) only occurred in 0–8% patients [14, 34, 35].

**Table 5:** Postoperative complications of published studies using INFIX

Variables	*N*	Wound issue	LFCN injury	FNI	HO	Fixation failure	Nonunion	INFIX discomfort	Other pain	TE	FU, month
Vaidya2012 [28]	91	3%	30%/1%	0	35%	3%	0	3%	0	0	15
Vaidya2012 [22]	22	0	9%	0	0	5%	0	5%	0	0	19
Müller2013 [21]	31	6%	19%/19%	0	29%	0	3%	0	0	0	53
Hoskins2016 [29]	21	7%	57%/57%	0	43%/5%	0	0	5%	0	10%	11
Shetty2017 [6]	15	7%	7%	0	0	13%	0	0	0	0	35
Fang2017 [30]	29	3%	48%	3%	0	7%	0	3%	10%	0	7
Dahill2017 [31]	47	2%	55%/34%	0	0	2%	0	0	0	0	38
Vaidya2017 [14]	83	4%	8%	0	68%/1%	1%	0	6%	0	0	35
Wu2018 [32]	23	0	13%	4%	35%	0	0	4%	9%	0	15
Li2019 [33]	28	0	21%	0	29%	0	0	7%	0	0	20
Hua2019 [34]	23	0	9%	0	0	0	0	0	0	0	14
Steer2019 [35]	24	8%	46%/25%	0	21%	4%	0	17%	0	17%	≥ 12
Vaidya2019 [36]	39	8%	0	0	10%	0	0	0	8%	0	22
Ebeed2020 [37]	16	0	19%	0	13%	0	0	0	0	6%	15
Du2020 [38]	17	6%	0	0	0	0	0	0	0	12%	7
Bi2017 [4]	21	0	14%	5%	0	0	0	0	0	0	17
Vaidya2017 [27]	24	4%	4%/4%	0	46%	8%	0	4%	0	0	40
Wang2017 [7]	26	0	8%	0	0	0	0	0	0	0	8
Ma2019 [8]	62	5%	0	0	0	0	0	0	0	0	≥12
Yin2019 [39]	35	3%	29%	0	34%	0	0	0	0	0	27
Current study	27	6%	4%	4%	0	4%	0	7%	7%	0	20
Case reports	8 femoral nerve palsies in 6 patients and a full recovery in 1 patient [40], 1 bladder incarceration [41], 1 vascular occlusion [42], 3 sciatic nerve palsies [36]										

*LFCN* lateral femoral cutaneous nerve, *FNI* femoral nerve injury, *HO* heterotopic ossification, *TE* thromboembolism, *FU* follow-up time

LFCN injury is the most common symptomatic INFIX complication and manifests as numbness, pain, and paresthesia. Most patients can spontaneously recover [32, 37] or gradually recover following the removal of INFIX [6, 14, 34]; however, 0–34% of patients were persistently affected (Table 5) [21, 31, 38]. In our study, 1 patient with unilateral LFCN injury recovered spontaneously after 2 weeks. Moreover, FN injury is one of the serious INFIX complications and manifests as numbness in the front of the thigh, weakness in the quadricep muscles, difficulty in walking, and weakness in knee extension [40]. Some patients gradually recovered after the removal of the INFIX or adjustment of the position in time [4, 32]. However, there are some who experienced permanent FN injury and complained of muscle weakness and numbness at the last follow-up [30, 40]. In this study, one patient with unilateral FN injury partially recovered after emergent INFIX adjustment and quadriceps strength was rated at 4/5 at the last follow-up.

The main reason for LFCN and FN injury is the absence of sufficient space between the titanium rod and bone surface of the ilium as well as the tissue under the screw head and rod [43]. Consequently, patients with obesity were more prone to FN injury owing to high abdominal pressure and excessive subcutaneous tissue [40]. Osterhoff et al. found in an autopsy study that INFIX was safe for LFCN, FN, and sartorius and rectus femoris when the rod-to-bone distance (RBD) was 20 mm [44]. Scherer et al. found that damage or irritation to LFCN would be reduced when RBD ranged from 20 to 25 mm and rod-to-symphysis distance (RSD) was within 40 mm [45]. In a study with an RBD of 5–10 mm, the incidence of LFCN injuries reached up to 55%, and 34% of the patients experienced persistent LFCN injuries [31], which may be caused by the RBD being far less than the aforementioned safe distance (RBD = 20 mm). However, RBD and rod contour were different in most published studies owing to the diverse habitus of patients and different surgical experience of surgeons in different regions (Table 5).

Moreover, LFCN had changeable courses (sartorius, posterior, and fan types) in the proximal thigh [46]. Careful operations are required because there is a high risk of LFCN injury from operation [47]. The specification of INFIX includes screw head diameter, screw length, screw head type, and connecting rod diameter. There were different specifications in published studies (Table 4), which may be related to neurovascular injury [29]. Meanwhile, some researchers believed that the excess rod at both ends could also cause LFCN injury and should be trimmed [33]. In summary, we suggest that taking the following actions is important to reduce the surgical injuries of INFIX: screws and connecting rods should be appropriately chosen based on the personal habitus of the patients, RBD and RSD should be given attention, and the amplitude of the peripheral nerves and vascular patency should be monitored if possible during the perioperative period. If signs and symptoms of nerve injury are noted postoperatively, emergency surgery is required to remove or adjust the internal fixation.

Although some studies reported heterotopic ossification (HO), deep vein thrombosis, vascular occlusion, bladder incarceration, chronic pain, and other injuries of pelvic organ (Table 5), these complications were not observed in this study. Two patients (7%) experienced rod-related discomfort, but it did not affect their daily activities. According to published studies and the current experience, the removal of INFIX after achieving bony union at 3–12 months postoperatively is recommended. This study first reported five patients with sacral nerve injury (four patients fully recovered, and one patient partially recovered).

This study has several limitations. First, only postoperative functional scores were recorded. Second, the follow-up period was not too long. Third, this was a single-center retrospective case series with inherent limitations of the study design, including not having a control group, standardized surgical indications, and a heterogeneous study population in terms of demographics and injury characteristics. Thus, multicenter, large-sample prospective controlled studies are important for further research on the therapeutic effect, postoperative complications, and technical modification of INFIX.

## Conclusions

INFIX combined with or without posterior SI screw can achieve satisfactory bony union and functional recovery for the treatment of unstable pelvic fractures, providing an alternative minimally invasive treatment for unstable pelvic injuries.

## Data Availability

The original data of this study are available from the corresponding author for reasonable request.

## Abbreviations

INFIX: Anterior subcutaneous internal fixation
EXFIX: External fixation
PDI: Pelvic deformity index
PSW: Pubic symphyseal width
SF-12: 12-Item Short-Form Health Survey
SI: Sacroiliac
ORIF: Open reduction and internal fixation
BMI: Body mass index
ISS: Injury severity score
ICU: Intensive care unit
MCS: Mental component score
PCS: Physical component score
SD: Standard deviation
IQR: Interquartile range
APC: Anteroposterior compression injuries
LC: Lateral compression injuries
VS: Vertical shear injuries
CM: Complex pelvic injuries
LFCN: Lateral femoral cutaneous nerve
FN: Femoral nerve
RBD: Rod-to-bone distance
RSD: Rod-to-symphysis distance
HO: Heterotopic ossification
CT: Computed tomography
